# Association between Sleep Duration, Physical Activity, and Mental Health Disorders: A Secondary Analysis of the National Survey of Children's Health 2017-2018

**DOI:** 10.1155/2021/5585678

**Published:** 2021-03-15

**Authors:** Shiting Xiang, Jie Dong, Xun Li, Liping Li

**Affiliations:** Hunan Children's Research Institute (HCRI), Hunan Children's Hospital, 86 Ziyuan Road, Changsha, China 410007

## Abstract

**Background:**

The purpose of this article was to examine the association of sleep duration and physical activity and their interactions on mental health disorders in American children aged 6-17 years.

**Methods:**

Data were analyzed from the combined 2017-2018 National Survey of Children's health. Ultimately, a total of 36370 children aged 6-17 years were selected as the samples. Weighted logistic regression models were used to estimate odds ratios and 95% confidence intervals.

**Results:**

Insufficient sleep duration was associated with an increased risk for current anxiety, depression, and behavior/conduct problems (odds ratio = 1.449, 1.991, 1.375; 95% confidence interval: 1.313-1.702, 1.648-2.406, 1.162-1.627). Insufficient physical activity was associated with an increased risk for current anxiety (odds ratio = 1.448; 95% confidence interval: 1.230-1.706) and depression (odds ratio = 1.743; 95% confidence interval: 1.304-2.329). In addition, additive interactions between sleep duration and physical activity were observed on current anxiety and depression.

**Conclusions:**

Insufficient sleep duration and insufficient physical activity in children were associated with mental health disorders. There is a synergistic interaction effect between insufficient sleep duration and insufficient physical activity on current anxiety and current depression.

## 1. Background

Mental health disorders account for 7.4 percent of the global disability-adjusted life years (DALYs) and 22.7 percent of the global disability-life years (YLDs) [1]. The leading two contributors are depression (9.6% of all YLDs) and anxiety (3.5% of all YLDs). Importantly, common mental disorders in adulthood usually first appear in childhood and adolescence, and about half of all lifetime mental disorders begin in midadolescence [2]. Estimates using nationally representative samples of US children have shown the prevalence of depression, anxiety, and conduct or behavior problems to be 3.2%, 7.1%, and 7.4%, respectively [3]. Therefore, early prevention of mental health problems among children and adolescents needs high priority.

A considerable amount of literature has shown that short sleep duration and low physical activity are associated with mental health disorders [4, 5]. Shorter sleep duration influencing mental health by disrupting the function of dorsal medial prefrontal cortex (dmPFC), which plays an important role in emotion control, has been proven by previous studies [6]. Lots of evidence supports that children and adolescents who engage in at least 60 minutes of physical activity a day benefit from reduced mental health disorders [7]. These evidences suggested prevention and early control of short sleep duration, and low physical activity may contribute to reducing the incidence of mental health disorders.

Although a consistent link between sleep, physical activity, and mental health disorders have been shown in most studies, the relationship between the interaction of these two factors and mental health disorder is not well characterized. Using physical and mental health data from the dataset of the National Survey of Children's Health (NSCH) 2017-2018, this study is aimed at examining the association and interaction between sleep duration, physical activity, and mental health disorders.

## 2. Methods

### 2.1. Study Population

This study is a secondary analysis using data from the 2017-2018 NSCH, and it is aimed at examining the association between sleep duration, physical activity, and mental health disorders. The NSCH is sponsored by the Maternal and Child Health Bureau and conducted by the National Center for Health Statistics. The survey is aimed at providing national-level and state-level data on the physical and mental health of children aged 0-17 years in the United States. The questionnaire was completed by mail and online. A child of the appropriate age was randomly selected from each eligible family for a detailed interview. The respondents were primary caregivers who had the best knowledge of children's health [8, 9].

We excluded samples from the analysis that did not have complete records, including key information like sleep duration, physical activity, anxiety problems, depression, and behavior/conduct problems. At last, a total of 36370 children aged 6-17 years were selected as the samples.

### 2.2. Measurement of Mental Health Disorders

In this study, current mental health disorders were measured using respondent reports in the NSCH survey to questions “Has a doctor or other health care provider ever told you that this child has anxiety problems?”, “Has a doctor or other health care provider ever told you that this child has depression?”, and “Has a doctor, other health care provider, or educator ever told you that this child has behavior/conduct problems?”. If yes, the following questions were asked: “Does this child currently have the condition?” A child was considered to have a current mental health disorder if there was a positive diagnosis of current anxiety, depression, and behavior/conduct problems.

### 2.3. Measurement of Sleep Duration

Data regarding sleep duration were obtained by respondent answers to the question “During the past week, how many hours of sleep did this child get on an average weeknight”; the NSCH classifies individuals' responses to this question into seven categories: less than 6 hours, 6 hours, 7 hours, 8 hours, 9 hours, 10 hours, and 11 or more hours. A dichotomous variable of sleep duration was determined if the child's sleep time was appropriate for age or not. The American Academy of Sleep Medicine has developed a guideline, which was utilized in the 2017-2018 NSCH, to recommend that children aged 6 to 12 should sleep 9 to 12 hours per 24 hours and children between 13 and 18 years old should sleep 8 hours to 10 hours every 24 hours [10].

### 2.4. Measurement of Physical Activity

Data on physical activity were measured by the respondent's answer to the question “During the past week, how many days did this child exercise, play a sport, or participate in physical activity for at least 60 minutes?” The NSCH classifies individuals' responses to this question into four categories: 0 days, 1-3 days, 4-6 days, and every day. A dichotomous category for physical activity was determined according to several global and national guidelines, which recommend that children accumulate at least 60 minutes of moderate to vigorous physical activity per day [7, 11].

### 2.5. Covariates

Covariates were selected from factors that were identified in previous publications as associated with mental health [3, 12]. Covariates were gender (male, female), race (Hispanic, non-Hispanic white, non-Hispanic black, other, or mixed race), highest adult education (less than high school, high school or GED, some college or technical school, college degree or higher), household poverty status (0-199%, 200-299%, 300-399%, ≥400%), and current health insurance status (insured, not insured).

### 2.6. Statistical Analysis

All statistical analyses were performed using IBM SPSS version 22.0 (IBM, New York, USA). Weighted population-based frequencies/percentages and 95% confidence interval (CI) for selected characteristics and outcomes were calculated using the weighting methods suggested by NSCH [13]. Between groups, comparisons were conducted using the Wald Chi-square test. Weighted logistic regression models were established to estimate odds ratios (OR). Additive interaction models were employed to examine the interactions between sleep duration and physical activity on each mental health disorder. All models were adjusted for gender, race, highest adult education, household poverty status, and current health insurance status. All test values were 2-sided, and *P* < 0.05 was considered significant.

## 3. Results

This study selected 36370 cases with complete information as the samples (weighted *n* = 48129281). The weighted estimates of percentages of current anxiety, depression, and behavior/conduct problems were 8.9% (95% CI: 8.4% ~9.4%), 3.9% (95% CI: 3.6% ~4.3%), and 7.5% (95% CI: 6.9% ~8.1%), respectively. About 64.8% of the children had slept age-appropriate hours in the past week, and about 22.6% had 60 minutes of physical activity every day during the past week.

Table 1 presents the weighted percentages and 95% CI for selected characteristics of study participants. The results of the *χ*^2^ test and significant differences of selected characteristics based on current mental health disorders are summarized in Table 1. Children with current mental health disorders had higher rates of sleep less than the recommended time than children without current mental health disorders (all *P* values<0.01). Children with current anxiety and current depression had higher rates of exercise less than the recommended time than children without current anxiety and current depression (all *P* values<0.01).

Table 2 shows unadjusted crude and adjusted odds ratios of sleep duration and physical activity for predicting each mental health disorders. Children who sleep less than the recommended time had higher unadjusted odds of all mental health disorders (all *P* values<0.05). Children who exercise less than the recommended time had higher unadjusted odds of all mental health disorders except for behavior/conduct problems (all *P* values<0.05). After adjusting for socio-demographic variables, the odds remain significant.

Table 3 shows the interaction between sleep duration and physical activity on mental health disorders. After adjusting for socio-demographic variables, children who sleep less than the recommended time and exercise less than recommended time had even higher adjusted odds of current anxiety and depression than either condition alone, indicating that there is an interaction between sleep duration and physical activity (all *P* values<0.05). However, no significant interaction was detected between sleep duration and physical activity on behavior/conduct problems.

## 4. Discussion

The purpose of this study was to examine the associations between sleep duration and physical activity on mental health disorders within a representative sample of US children and adolescents. In this study, we revealed that both insufficient sleep duration and insufficient physical activity were associated with mental health disorders, while insufficient physical activity was not associated with behavior/conduct problems. Our study has also shown that sleep time deficits and physical activity deficits have an interactive impact on current anxiety and depression.

Our study found a higher prevalence of anxiety (8.9%) than those reported previously from other surveys [3, 12, 14]. The prevalence of depression and behavior/conduct problems was similar to a previous study using data from the 2016 NSCH, which showed that 7.4% had a current behavior/conduct problem and 3.2% had current depression among children aged 3–17 years [3]. Consistent with previous researches, anxiety and behavior/conduct problems are more common than depression [3, 15]. The percentage of children meeting the sleep recommendations (64.8%) and the percentage meeting the physical activity recommendations (22.6%) differed from previous studies. For example, data from the 2016-2017 NSCH reported that 19.3% of children aged 10-17 years in the United States had been physically active for a total of 60 minutes per day [16]. These differences may be due to differences in the age groups studied. The participants in our study ranged in age from 6 to 17 years old and were different from most other surveys.

Previous reports have already shown that insufficient sleep duration can increase the risk of many diseases, such as cardiovascular and metabolic disease risk [17, 18], neurocognitive dysfunction [19], and mental health [20]. Our study confirmed that insufficient sleep time was associated with an increased risk of current anxiety, depression, and behavioral/conduct problems, even after full adjustment for potential confounding factors. Reviews of various studies have suggested that participation in physical activity can improve children and adolescents' mental health by distracting from negative rumination and helping to produce beneficial and mood-elevating neurotransmitters [21, 22]. Our results also showed insufficient physical activity increased the risk of current anxiety and depression, which was consistent with previous studies.

Extensive studies have been carried out regarding the relationship between sleep and physical activity on mental health disorders, but few studies have focused on their interaction on mental health disorders. A new finding from our study is a synergistic interaction effect between insufficient sleep duration and insufficient physical activity on current anxiety and current depression. Children with both insufficient sleep duration and physical activity are at significantly increased risk of anxiety and depression disorders than either condition alone.

One of the main strengths of this study is that we included a representative sample of US children and adolescents to investigate the relationship between sleep and physical activity on mental health disorders. Furthermore, we used the latest data that had been consolidated for two years. The study provides more evidence for further studies to demonstrate a correlation between sleep duration and physical activity with mental health disorders.

However, the research does have limitations. First, data from the NSCH is based on parent self-report, with the possibility of information bias. The association between sleep duration, physical activity, and mental health disorders may lead to a bit deviation because of the potential inaccuracy of information. Second, the sleep variable only asked about sleep duration; detailed information was not reported such as sleep quality. The same, physical activity variable contained only frequency, not information such as activity intensity. Third, measurements of sleep duration and physical activity were based on parents' self-reports rather than objective activity recorder measurements. Using self-reported sleep duration and physical activity might introduce some measurement error and result in information bias of association between sleep duration, physical activity, and mental health disorders. Besides, the NSCH data were cross-sectional which did not allow us to make a causal inference. Therefore, all relationships are related, and further prospective research should be done to overcome this methodological limitation.

## 5. Conclusion

Insufficient sleep duration and insufficient physical activity in children were associated with mental health disorders. There is a synergistic interaction effect between insufficient sleep duration and insufficient physical activity on current anxiety and current depression.

## Figures and Tables

**Table 1:** Weighted percentages and 95% CI for selected characteristics of children aged 6-17 years based on current mental health disorders, 2017-2018 NSCH.

Characteristics	Current anxiety			Current depression			Current behavior/conduct problems		
	No	Yes	*P*	No	Yes	*P*	No	Yes	*P*
	*n* = 32114	*n* = 4256		*n* = 34468	*n* = 1902		*n* = 33505	*n* = 2865	
	% (95% CI)^a^	% (95% CI)^a^		% (95% CI)^a^	% (95% CI)^a^		% (95% CI)^a^	% (95% CI)^a^	
Age (years)									
6-12	59.4 (58.2, 60.6)	48.4 (45.5, 51.3)	<0.001	59.6 (58.5, 60.7)	29.8 (25.6, 34.5)	<0.001	57.8 (56.7, 59.0)	66.0 (62.4, 69.5)	<0.001
13-17	40.6 (39.4, 41.8)	51.6 (48.7, 54.5)		40.4 (39.3, 41.5)	70.2 (65.5, 74.4)		42.2 (41.0, 43.3)	34.0 (30.5, 37.6)	
Gender									
Male	51.5 (50.3, 52.8)	47.7 (44.8, 50.6)	0.017	51.5 (50.3, 52.7)	44.0 (39.6, 48.4)	0.001	50.0 (48.8, 51.2)	66.1 (62.1, 69.8)	<0.001
Female	48.5 (47.2, 49.7)	52.3 (49.4, 55.2)		48.5 (47.3, 49.7)	56.0 (51.6, 60.4)		50.0 (48.8, 51.2)	33.9 (30.2, 37.9)	
Race									
Hispanic	26.3 (25.1, 27.7)	18.4 (15.6, 21.6)	<0.001	25.9 (24.7, 27.2)	18.7 (14.9, 23.2)	0.003	26.0 (24.8, 27.4)	20.7 (16.9, 25.1)	<0.001
Non-Hispanic white	48.9 (47.7, 50.1)	65.7 (62.6, 68.8)		50.0 (48.9, 51.2)	59.2 (54.5, 63.8)		50.3 (49.2, 51.5)	51.5 (47.6, 55.4)	
Non-Hispanic black	14.4 (13.5, 15.3)	8.3 (6.7, 10.4)		13.9 (13.1, 14.8)	12.6 (9.6, 16.4)		13.3 (12.5, 14.2)	20.1 (16.8, 24.0)	
Other/mixed race	10.4 (9.8, 11.0)	7.5 (6.3, 8.9)		10.1 (9.6, 10.7)	9.4 (7.1, 12.5)		10.3 (9.7, 10.9)	7.7 (6.3, 9.4)	
Highest adult education									
Less than high school	10.9 (9.8, 12.1)	6.9 (5.0, 9.3)	0.006	10.6 (9.6, 11.8)	7.8 (5.3, 11.3)	<0.001	10.5 (9.5, 11.7)	10.4 (7.1, 15.1)	<0.001
High school or GED	19.7 (18.6, 20.7)	19.2 (16.7, 21.9)		19.5 (18.5, 20.5)	23.5 (19.5, 27.9)		19.2 (18.2, 20.2)	25.2 (21.9, 28.8)	
Some college or technical school	22.1 (21.2, 23.0)	24.1 (21.9, 26.6)		21.9 (21.0, 22.8)	30.4 (26.5, 34.6)		21.9 (21.0, 22.8)	27.2 (24.2, 30.4)	
College degree or higher	47.4 (16.2, 48.6)	49.8 (46.9, 52.7)		48.0 (46.9, 49.1)	38.4 (34.3, 42.6)		48.5 (47.3, 49.6)	37.2 (33.8, 40.7)	
Household poverty status									
0-199%	41.5 (40.3, 42.8)	40.3 (37.4, 43.3)	0.805	41.0 (39.8, 42.3)	50.8 (46.4, 55.2)	<0.001	40.4 (39.1, 41.6)	54.5 (50.7, 58.2)	<0.001
200-299%	15.2 (14.4, 16.0)	16.1 (14.0, 18.5)		15.4 (14.6, 16.2)	12.9 (10.6, 15.7)		15.4 (14.6, 16.2)	14.5 (12.0, 17.3)	
300-399%	11.9 (11.2, 12.6)	11.9 (10.3, 13.8)		12.0 (11.4, 12.7)	8.7 (6.7, 11.2)		12.1 (11.4, 12.8)	9.9 (8.2, 12.1)	
≥400%	31.3 (30.4, 32.3)	31.6 (29.2, 34.0)		31.5 (30.6, 32.5)	27.6 (23.9, 31.5)		32.2 (31.2, 33.2)	21.1 (18.7, 23.6)	
Health insurance status									
Insured	92.8 (91.9, 93.5)	94.7 (92.6, 96.3)	0.082	92.8 (92.0, 93.6)	95.8 (93.4, 97.3)	0.020	92.8 (92.0, 93.6)	94.2 (92.2, 95.7)	0.188
Not insured	7.2 (6.5, 8.1)	5.3 (3.7, 7.4)		7.2 (6.4, 8.0)	4.2 (2.7, 6.6)		7.2 (6.4, 8.0)	5.8 (4.3, 7.8)	
Sleep duration									
Recommended	65.5 (64.3, 66.7)	57.7 (54.7, 60.6)	<0.001	65.4 (64.2, 66.5)	50.5 (46.0, 54.9)	<0.001	65.6 (64.4, 66.7)	55.2 (51.2, 59.1)	<0.001
<Recommended	34.5 (33.3, 35.7)	42.3 (39.4, 45.3)		34.6 (33.5, 35.8)	49.5 (45.1, 54.0)		34.4 (33.3, 35.6)	44.8 (40.9, 48.8)	
Sleep duration (hours)									
<6	1.0 (0.8, 1.3)	5.0 (3.7, 6.7)	<0.001	1.0 (0.8, 1.2)	10.0 (7.4, 13.5)	<0.001	1.1 (0.9, 1.3)	4.9 (3.5, 6.7)	<0.001
6	3.6 (3.1, 4.1)	6.5 (5.0, 8.5)		3.6 (3.1, 4.1)	10.4 (7.6, 14.2)		3.7 (3.2, 4.2)	5.8 (4.3, 7.8)	
7	12.2 (11.4, 13.0)	16.2 (14.0, 18.6)		12.2 (11.5, 13.0)	20.7 (17.3, 24.6)		12.5 (11.8, 13.3)	12.8 (10.3, 15.8)	
8	35.4 (34.3, 36.6)	32.3 (29.8, 35.0)		35.4 (34.2, 36.5)	30.2 (26.5, 34.1)		35.3 (34.2, 36.5)	33.0 (29.2, 37.1)	
9	28.2 (27.1, 29.3)	22.2 (20.1, 24.5)		28.1 (27.0, 29.1)	17.4 (14.6, 20.5)		27.9 (26.9, 29.0)	24.0 (21.3, 27.0)	
10	16.6 (15.7, 17.4)	14.5 (12.5, 16.7)		16.8 (16.0, 17.6)	7.0 (5.1, 9.4)		16.4 (15.6, 17.3)	15.5 (13.3, 18.0)	
>10	3.1 (2.7, 3.5)	3.2 (2.2, 4.6)		3.0 (2.7, 3.4)	4.4 (2.4, 7.9)		3.0 (2.7, 3.4)	4.0 (2.7, 5.7)	
Physical activity									
Recommended	23.2 (22.2, 24.2)	16.2 (14.2, 18.3)	<0.001	23.0 (22.0, 24.0)	12.7 (9.8, 16.2)	<0.001	22.4 (21.4, 23.4)	24.9 (22.0, 28.2)	<0.001
<Recommended	76.8 (75.8, 77.8)	83.8 (81.7, 85.8)		77.0 (76.0, 78.0)	87.3 (83.8, 90.2)		77.6 (76.6, 78.6)	75.1 (71.8, 78.0)	
Physically active days out of 7 days^b^									
0 day	8.5 (7.9, 9.2)	18.3 (16.0, 20.9)	<0.001	8.8 (8.1, 9.5)	24.6 (21.0, 28.4)	<0.001	8.8 (8.2, 9.5)	16.3 (13.2, 20.0)	0.112
1-3 days	39.7 (38.5, 40.9)	42.6 (39.8, 45.5)		39.8 (38.6, 40.9)	45.4 (40.9, 49.9)		40.2 (39.0, 41.4)	37.6 (34.0, 41.3)	
4-6 days	28.6 (27.6, 29.6)	23.0 (20.6, 25.4)		28.5 (27.5, 29.5)	17.4 (14.3, 21.1)		28.6 (27.6, 29.6)	21.2 (18.2, 24.6)	
Everyday	23.2 (22.2, 24.2)	16.2 (14.2, 18.3)		23.0 (22.0, 24.0)	12.7 (9.8, 16.2)		22.4 (21.4, 23.4)	24.9 (22.0, 28.2)	

^a^Weighted estimates of percentages and 95% CI.

**Table 2:** Logistic regression of associations between sleep duration, physical activity, and current mental health disorders of children aged 6-17 years, 2017-2018 NSCH.

Factors	Current anxiety		Current depression		Current behavior/conduct problems	
	OR (95% CI)	OR (95% CI)^a^	OR (95% CI)	OR (95% CI)^a^	OR (95% CI)	OR (95% CI)^a^
Sleep duration						
Recommended	1 (reference)	1 (reference)	1 (reference)	1 (reference)	1 (reference)	1 (reference)
<Recommended	1.391 (1.219, 1.588)	1.495 (1.313, 1.702)	1.853 (1.537, 2.233)	1.991 (1.648, 2.406)	1.545 (1.308, 1.826)	1.375 (1.162, 1.627)
Physical activity						
Recommended	1 (reference)	1 (reference)	1 (reference)	1 (reference)	1 (reference)	1 (reference)
<Recommended	1.563 (1.330, 1.837)	1.448 (1.230, 1.706)	2.054 (1.535, 2.750)	1.743 (1.304, 2.329)	0.867 (0.726, 1.034)	0.970 (0.811, 1.162)

^a^Adjusting for age, gender, race, highest adult education, household poverty status, and current health insurance status.

**Table 3:** Estimation of combined effects of sleep duration and physical activity on current mental health disorders risk of children aged 6-17 years, 2017-2018 NSCH.

Sleep duration	Physical activity	Current anxiety		Current depression		Current behavior/conduct problems	
		OR (95% CI)	OR (95% CI)^a^	OR (95% CI)	OR (95% CI)^a^	OR (95% CI)	OR (95% CI)^a^
Recommended	Recommended	1 (reference)	1 (reference)	1 (reference)	1 (reference)	1 (reference)	1 (reference)
Recommended	<Recommended	1.548 (1.263, 1.899)	1.429 (1.166, 1.753)	2.226 (1.485, 3.335)	1.888 (1.263, 2.824)	0.847 (0.680, 1.054)	0.952 (0.765, 1.186)
<Recommended	Recommended	1.386 (1.025, 1.873)	1.508 (1.111, 2.047)	2.204 (1.270, 3.824)	2.463 (1.397, 4.343)	1.543 (1.134, 2.100)	1.382 (1.008, 1.895)
<Recommended	<Recommended	2.126 (1.703, 2.654)	2.098 (1.680, 2.620)	3.941 (2.604, 5.964)	3.563 (2.339, 5.427)	1.318 (1.032, 1.685)	1.312 (1.023, 1.681)

^a^Adjusting for age, gender, race, highest adult education, household poverty status, and current health insurance status.

## Data Availability

The NSCH data and the permission for its use were obtained from a request from the Data Resource Center for Child and Adolescent Health (https://www.childhealthdata.org/dataset). More details regarding NSCH data availability and ethical standards are available at https://www.childhealthdata.org.
